# Influences of climate change on area variation of Qinghai Lake on Qinghai-Tibetan Plateau since 1980s

**DOI:** 10.1038/s41598-018-25683-3

**Published:** 2018-05-09

**Authors:** Lingyi Tang, Xiaofang Duan, Fanjin Kong, Fan Zhang, Yangfan Zheng, Zhen Li, Yi Mei, Yanwen Zhao, Shuijin Hu

**Affiliations:** 1 College of Resources and Environmental Sciences, Nanjing Agricultural University, Nanjing, Jiangsu 210095 China; 2 MLR Key laboratory of Saline Lake Resources and Environment, Institute of Mineral Resources, Chinese Academy of Geological Science (CAGS), Beijing, 100037 China; 3 State Key Laboratory for Mineral Deposits Research, Nanjing University, Nanjing, Jiangsu 210046 China; 4 School of Water Resources and Environment, China University of Geosciences (Beijing), Beijing, 100083 China; 5 Department of Entomology & Plant Pathology, North Carolina State University, Raleigh, NC 27695 USA

## Abstract

Qinghai-Tibetan Plateau is the most sensitive region to global warming on Earth. Qinghai Lake, the largest lake on the plateau, has experienced evident area variation during the past several decades. To quantify the area changes of Qinghai Lake, a satellite-based survey based on Landsat images from the 1980s to 2010s has been performed. In addition, meteorological data from all the seven available stations on Qinghai-Tibetan Plateau has been analyzed. Area of Qinghai Lake shrank ~2% during 1987–2005, and then increased ~3% from 2005–2016. Meanwhile, the average annual temperature increased 0.319 °C/10 y in the past 50 years, where the value is 0.415 °C/10 y from 2005–2016. The structural equation modeling (SEM) shows that precipitation is the primary factor influencing the area of Qinghai Lake. Moreover, temperature might be tightly correlated with precipitation, snow line, and evaporation, thereby indirectly causes alternations of the lake area. This study elucidated the significant area variation of water body on the Qinghai-Tibetan Plateau under global warming since 1980s.

## Introduction

**Figure 1:**
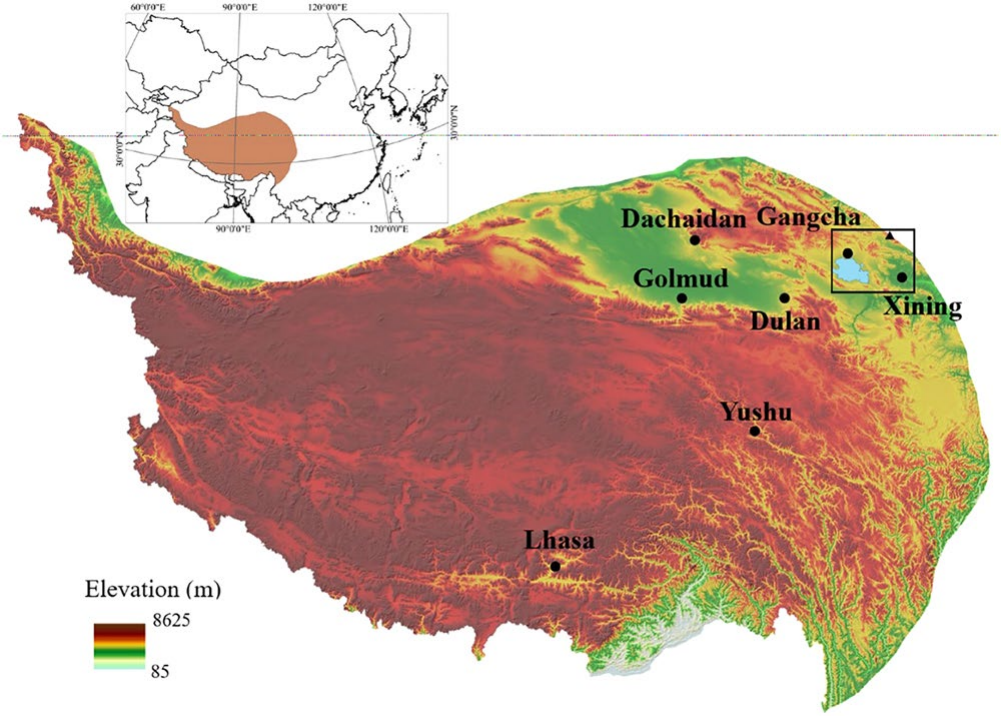
The distributions of all the seven meteorological stations (Dachaidan, Dulan, Gangcha, Golmud, Xining, Yushu, and Lhasa). Gangshika peak is marked as triangle. The image was generated by ArcGIS software (Version 10.2 for Desktop).

Qinghai-Tibetan Plateau is the largest plateau with the highest average altitude on the planet. It is also the most sensitive area on Earth^1^. In addition, it is highly influenced by the East Asian monsoon, the Indian monsoon, and the Westerly^2–4^. Qinghai Lake (N36.51°–37.25°; E99.58°–100.79°) is located in northeastern Qinghai-Tibetan Plateau, and it is the largest lake in China (see Fig. 1). It is a salt lake with the lake area of ~4300 km^2^. The lake is surrounded by Qilian Mountains, AltynTagh Mountains, and the Kunlun Mountains. Gangshika peak (N101.51°, E37.69°, 5254.5 m) of Qilian Mountains is ~120 km away from Qinghai Lake, which is the peak nearest to Qinghai Lake with altitude >5000 m.

The annual mean temperature of the Qinghai-Tibetan Plateau has increased by 0.27 °C/10 y during 1961–2005^5^. The increasing rate is higher than global mean surface temperature warming, i.e., 0.85 °C since 1880^6^. It was also proposed that over 7000 glaciers in Qinghai-Tibetan Plateau have been shrunk due to global warming^7^. Fluctuation of discharge of rivers^8,9^, as well as geologic hazards resulting from glaciers changes^10^, hence frequently occurs.

Previous studies have proposed that lakes are sensitive to global climate change and reflect alternations of surrounding environment^11–13^. The surface area of lakes, including the lakes in Qinghai-Tibetan Plateau, is a reliable indicator of climate change^3,14^. However, geological resources in many lakes on the Qinghai-Tibetan Plateau have been exploited, which dramatically influences the shape and area of the lakes^15^. Moreover, the development of agriculture and reclaiming farming resulted in the shrinking of Qinghai Lake during the 1950s to 1960s^3^. To the contrast, Qinghai Lake had been managed as Qinghai Lake National Nature Reserve since 1975, which avoids anthropogenic changes of the lakes. Therefore, Qinghai Lake is an ideal candidate to explore the responses of waterbody alternations to climate variations.

The aim of this study is to investigate the correlations among climate change, average altitude of snow line, and the area of Qinghai Lake based on spatial analysis of remotely sensed imagery and meteorological data. The remote sensing images of Qinghai Lake during 1987–2016 were collected to calculate its area. Meanwhile, the meteorological data of Qinghai-Tibetan Plateau during 1970–2016 was collected and analyzed.

## Results

### Climate fluctuation

**Figure 2:**
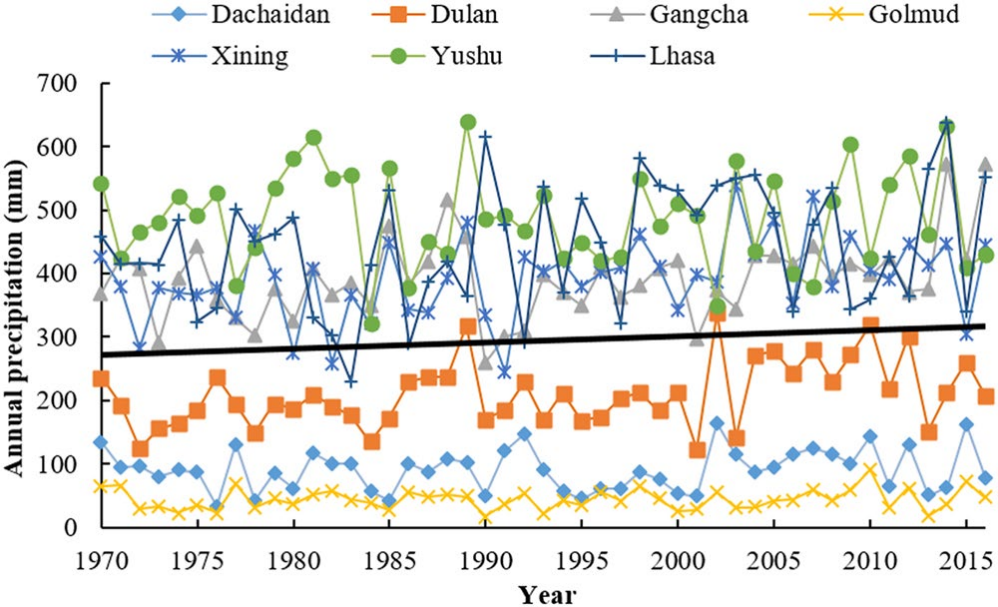
Annual precipitation of Dachaidan, Dulan, Gangcha, Golmud, Xining, Yushu and Lhasa from 1970–2016. The black solid line is the trend line of average values of annual precipitation for the seven locations.

Between 1970 and 2015, annual precipitation for the seven meteorological stations in Qinghai-Tibetan Plateau increased by 10.7 mm per decade. Compared with temperature, precipitation variation on Qinghai-Tibetan Plateau had evidently spatial heterogeneity. Annual precipitation variedamong the seven stations, e.g., the annual precipitation changes of Dachaidan, Dulan, Gangcha, Golmud, Xining, Yushu and Lhasa were 3.295, 15.38, 15.603, 1.452, 1.754, −1.269 and 19.271 mm per decade respectively (Fig. 2). Lhasa and Gangcha had the largest increase of annual precipitations among all the meteorological stations. From 2005 to 2016, annual precipitations of Dachaidan, Dulan, Gangcha, Golmud, Xining, Yushu and Lhasa had the changes of −17.063, −52.598, 81.262, −0.521, −44.395, 13.199 and 70.927 mm/10 y respectively. The precipitation in Gangcha meteorological station hence had the largest value. Moreover, average annual precipitation in Gangcha during 2005–2016 was 313.53 mm, with an elevation of 26.26 mm than that during 1970–2004.

**Figure 3:**
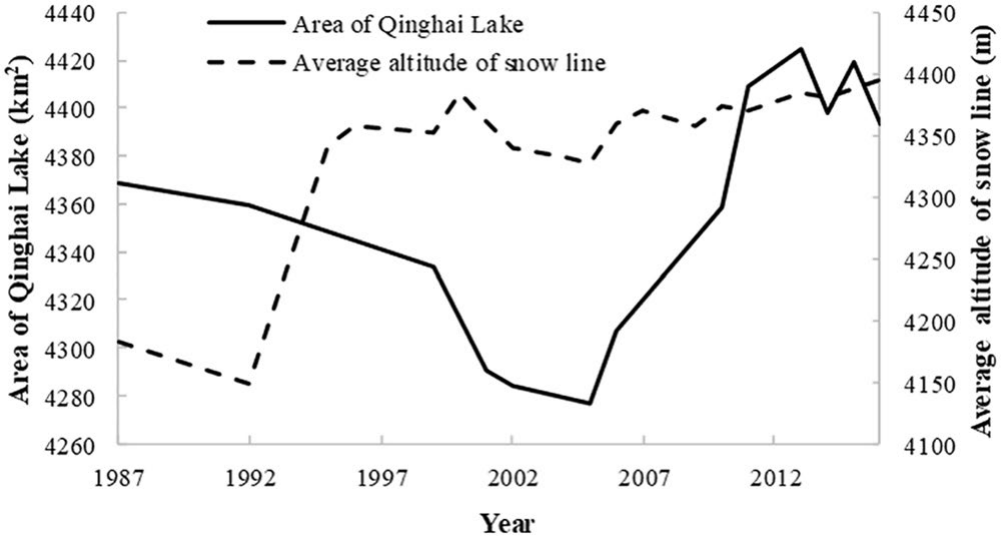
Area of Qinghai Lake in July and August and average altitude of snow line in Gangshika peak ranged from 1987–2016.

Glaciers are of considerable interest because of their high sensitivity to global warming^15^. Remote sensing images around August were selected to study the changes from 1987 to 2016. According to Fig. 3, the average altitude of snow line of Gangshika peak declined from 4342.2 to 4326.9 m during 1995–2005. However, from 2006–2016, the snow line was elevated from 4360.2 to 4385.3 m due to snow melting.

**Figure 4:**
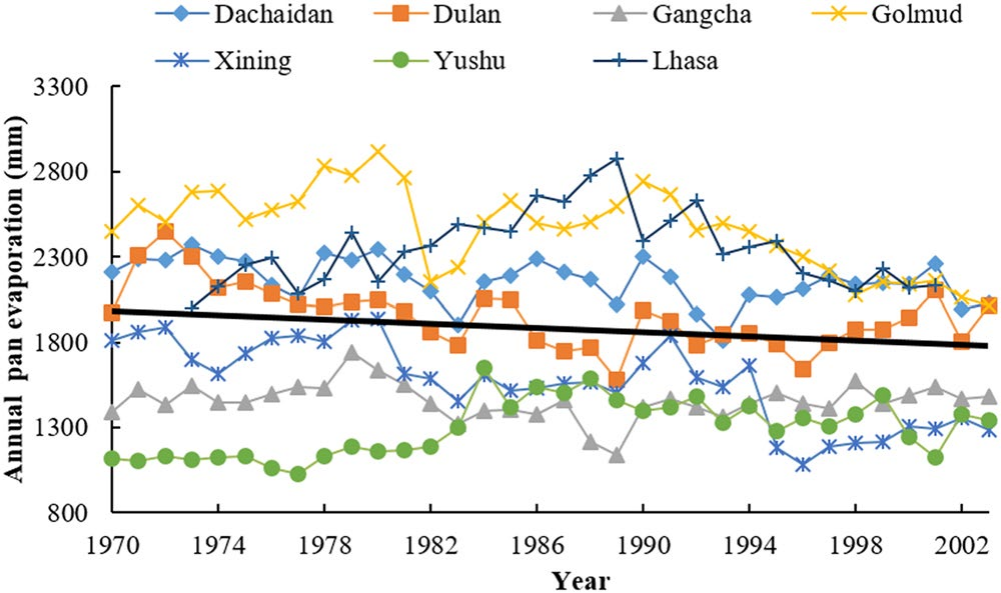
Annual pan evaporation of Dachaidan, Dulan, Gangcha, Golmud, Xining, Yushu and Lhasa from 1970–2003 measured by small evaporator. The black solid line is the trend line of the average values of annual pan evaporation collected from the seven locations.

**Figure 5:**
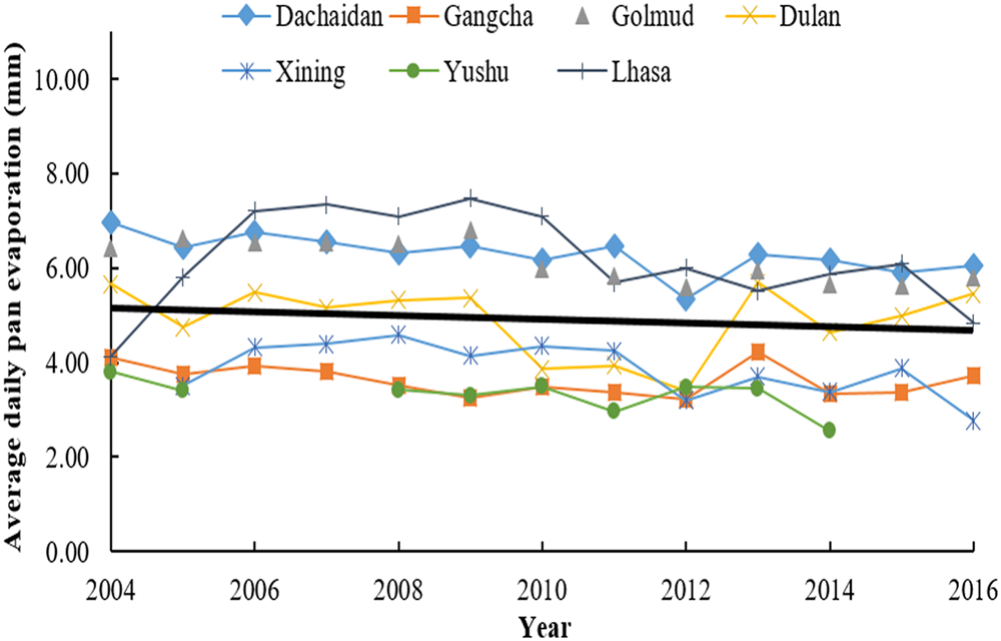
Average daily pan evaporation of Dachaidan, Dulan, Gangcha, Golmud, Xining, Yushu, and Lhasa from June to August within 2004–2016 (no available data of Yushu in 2006–2007 and 2015–2016). The evaporation was measured by evaporation tank. The black solid line is the trend line of the average values of average daily pan evaporation collected from the seven locations.

Evaporation is an important factor related to water budget on Qinghai-Tibetan Plateau, which is affected directly by climate change^3^. Annual pan evaporation of Dachaidan, Dulan, Gangcha, Golmud, and Xining has decreased by 6.255, 11.059, 1.343, 16.21 and 19.433 mm/y respectively during the period of 1970–2003 (Fig. 4). However, annual pan evaporation of Yushu and Lhasa increased by 9.089 and 1.634 mm/y. The average value of annual pan evaporation of all stations was −6.34 mm/y. During the period of 2004–2016, average daily evaporation from June to August of the seven locations decreased by 0.5–0.9 mm/10 y, including Yushu and Lhasa (the measuring methods were different due to the change of evaporator) (Fig. 5).

**Figure 6:**
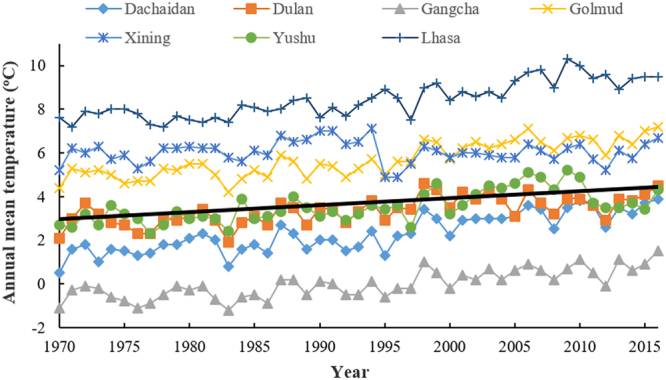
Annual mean temperature of Dachaidan, Dulan, Gangcha, Golmud, Xining, Yushu and Lhasa from 1970–2016. The black solid line is the trend line of average values of annual mean temperature collected from the seven locations.

During the period of 1970–2015, the average value of annual mean temperature in the seven meteorological stations increased 0.319 °C per decade. Furthermore, the temperatures of all stations increased to different extents. The annual mean temperature changes of Dachaidan, Dulan, Gangcha, Golmud, Xining, Yushu, and Lhasa were 0.542, 0.305, 0.371, 0.489, 0.014, 0.358 and 0.514 °C per decade respectively (Fig. 6). Xining station shows the lowest increase of temperature in the past 50 years, probably due to that it is on the edge of the plateau.

### Dynamic changes of Qinghai Lake area and SEM results

Remote sensing images in the period of mid-July to mid-August were further selected to study the changes among years from 1987 to 2016. In Table 1, Qinghai Lake’s area has fluctuated from 1987 to 2016. In the period of 1987–2005, Qinghai Lake shrank 91.82 km^2^ constantly. Then, it increased by 147.8 km^2^ from 2005 to 2013 constantly. From 2013 to 2016, the lake area fluctuated with the small variation of ~30 km^2^.

**Table 1:** Area of Qinghai Lake from 1987 to 2016 calculated by remote sensing data.

Year	Month	Area (m^2^)
1987	8	4368519000
1992	8	4359278700
1995	8	4345330500
1999	8	4333906800
2001	7	4290741900
2002	7	4284276300
2005	7	4276696500
2006	8	4307135400
2010	7	4358232900
2011	7	4409311500
2013	8	4424501248
2014	7	4397698800
2015	7	4419206100
2016	7	4393734144

Images in 2013 and 2016 were selected from Gaofen-1 WFV4, other years were based on Landsat TM/ETM+/OLI. Data of the least clouds interference was selected in July or August (obvious alternations of snow line).

The rationality test of SEM includes coefficient rationality, significance test, and goodness of fit test. The overall fitness of the model is expressed by normed fit index (NFI), Goodness of fit index (GFI), Comparative fit index (CFI), Incremental fit index (IFI), and Root mean square error of approximation (RMSEA). Fitting results of the model are given directly by AMOS software (Table 2). It indicates the model fitted well, and the relationships among the lake area, temperature, precipitation, evaporation, and snow line can be well reflected.

**Table 2:** Goodness of fit index for SEM model (the model of relationships among Qinghai Lake area, temperature, precipitation, evaporation, and snow line).

Index	χ^2^/df	GFI	NFI	CFI	RMSEA
Evaluation Criterion	<3	>0.9	>0.9	>0.9	<0.1
Result	1.04	0.97	0.92	0.97	0.02

**Figure 7:**
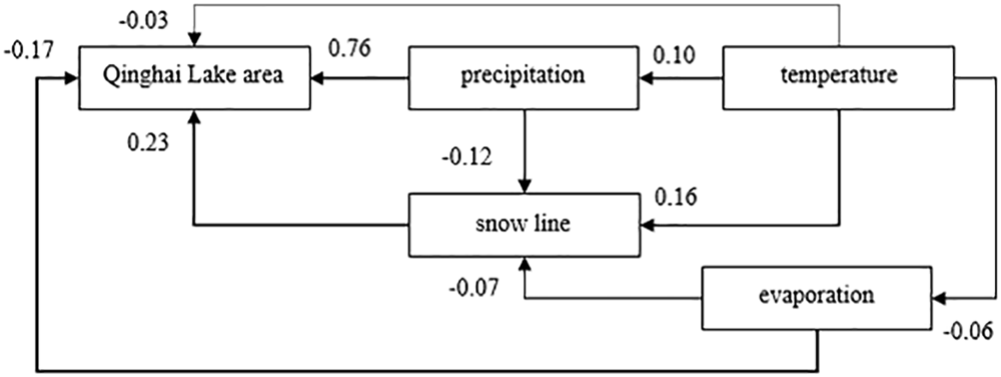
A model of relationships between Qinghai Lake area, temperature, precipitation, evaporation and snow line. Data of Gangcha station that nearest to Qinghai Lake is selected (Thickness of the line indicates the strength of the correlation, and the number represents the correlation coefficient. The dotted line indicates that the correlation is not significant).

The maximum likelihood method was applied to evaluate the model. As shown in Fig. 7, the lake area is affected by precipitation, snow line, evaporation, and temperature. It confirmed a strong positive correlation between the precipitation and lake area (coefficient =0.76, P = 0.025). Snow line and lake area are also positively related (coefficient = 0.23, P = 0.036), while both evaporation and temperature have evidently negative correlation with lake area (coefficient = −0.17, P = 0.049; coefficient = −0.03, P = 0.065).

Temperature has a certain impact on precipitation, snow line, and evaporation (coefficient = 0.10, P = 0.043; coefficient = 0.16, P = 0.010; coefficient = −0.06, P = 0.020). Moreover, effect of precipitation on the snowline around Qinghai Leke is notable (coefficient = −0.12, P = 0.012), only second to temperature. Furthermore, there is a weakly negative correlation between the evaporation and snow line (coefficient = −0.07, P = 0.030).

## Discussion

Change of lake area is tightly related to climate and environment evolution^16,17^. The alternations of the Qinghai Lake area, ideally, mostly reflect the natural processes^17^. Precipitation, evaporation, temperature, and snow line are common climatic factors, which affect the area of Qinghai Lake more directly than other natural factors in a short term^17,18^, i.e., since 1980s in this study. Climatic characteristics of the Qinghai-Tibetan Plateau are heterogeneous based on seven meteorological stations, excluding temperature. This confirms the sensitivity Qinghai-Tibetan Plateau to global warming.

The area of Qinghai Lake had shrunk slowly in the period of 1987 to 2005. In the downward trend of lake area from 1987–2005, precipitation increased slightly, along with lower snow line and quite higher evaporation. Then, a rapid increase of lake area was observed from 2005–2016. Within this period, it also had increasing precipitation, but with elevated snow line and reduced evaporation. In addition, there is a certain fluctuation of lake area from 2014 to 2016, which is consistent with the precipitation changes (also with evident fluctuation) in Gangcha station (the nearest meteorological station to Qinghai Lake). Therefore, the change of the lake area on the plateau should be attributed to combined climatic variables. As an important response area of global climate change, the temperature was rising in Qinghai-Tibetan Plateau, especially in recent years, which has also been revealed in the previous studies^5,19–22^. Moreover, although temperature has no significantly direct influence on the area of Qinghai Lake, it drives the changes of snow line, thereby affecting the lake area. Furthermore, evaporation usually contributes to outflow of lakes, but it has the low magnitude on the plateau. It is also possible that the vegetation and wind, in addition to water bodies, could influence evaporation. Therefore, evaporation has limited effect on the area of Qinghai Lake based on our results.

As the SEM model shows that precipitation is the primary factor of Qinghai Lake area variation. It is consistent with the previous study^23^, which reconstructed the fluctuation of lake level in the last 600 years and the results showed that there was a tight relationship between lake level and precipitation. Rivers, whose runoff due to glaciers and precipitation, plays important roles in the supply of natural water of Qinghai Lake. Although surface runoff accounts for 46.59% of the water in Qinghai Lake, D-O water isotope experiments had demonstrated that the primary source of runoff was precipitation, not glacial meltwater^18^. Therefore, although evaporation, snow line, and temperature have certain influences on Qinghai Lake area, the primary driver of the change in Qinghai Lake area is the increase of precipitation. Meanwhile, all the available data of remote sensing images and meteorological information related to the research were collected and analyzed. More available data, including both remote sensing and climatic data, would promote our further understanding of the global climate changes on the Qinghai-Tibetan Plateau.

## Methods

### Data collection

Meteorological data of all available bases of China Meteorological Network (http://data.cma.cn/) in Qinghai-Tibetan Plateau was collected, i.e., Dachaidan, Dulan, Gangcha, Golmud, Xining, Yushu, and Lhasa. The locations of the seven stations are shown in Fig. 1 and Table 3. Annual mean temperature and annual precipitation data were collected from 1970 to 2015 (all the available data). In addition, evaporation data from 1970 to 2016 were also analyzed. From 1970 to 2003, annual pan evaporation was provided. After 2003, only daily evaporation was available and different evaporator were applied. Thus, average daily evaporation from June to August after 2003 was selected to match the following GIS data.

**Table 3:** Coordinates of the seven available meteorological stations on Qinghai-Tibetan Plateau.

Location	Province	Longitude (N)	Latitude (E)
Dachaidan	Qinghai	37°51′16.98″	95°21′15.95″
Dulan	Qinghai	36°18′14.09″	98°05′40.51″
Gangcha	Qinghai	37°19′40.38″	100°08′30.22″
Golmud	Qinghai	36°24′10.46″	94°54′08.62″
Xining	Qinghai	36°37′02.27″	101°46′33.24″
Yushu	Qinghai	33°00′26.49″	97°00′22.00″
Lhasa	Tibet	29°38′48.50″	91°06′46.58″

Landsat TM/ETM+/OLI images covering the studied area from 1987 to 2015 were selected, which were downloaded from USGS website (http://landsat.usgs.gov/). Data of several years cannot be obtained due to cloud interference, which is shown in Table 1. Snow line of Gangshika peak was analyzed by GIS images during July-August, when Qinghai has the highest temperature. Two Gaofen-1 WFV4 images were selected due to the lack of appropriate images of the lake (year 2013 and 2016), which can be gained from RS Cloud Mart (http://www.rscloudmart.com/en). Additionally, remote sensing images of all months were selected to study the changes of Qinghai Lake for reference and calibration.

### Data analysis

EVNI (Version 5.0) and ArcGIS software (Version 10.2 for Desktop) were applied to process the Landsat TM/ETM+/OLI images (L1T) for lake area calculating. Modification of the normalized difference water index (MNDWI)^24^ and reclassification were selected to calculate the area of Qinghai Lake by Landsat TM/ETM+/OLI images. Gaofen-1 WFV4 images require pretreatment, including radiometric calibration, ortho-rectification, and atmospheric correction. Band ratio (band 2/band 4) was applied to calculate the area via GF-1 WFV4 images.

Bands 5, 4, and 3 (for R, G and B respectively) of Landsat TM/ETM+ were selected for false color composites (bands 6, 4, and 3 for Landsat8 OLI), so that snow and glaciers could be differentiated. More than 20 points were selected along the snow line of Lenglong mountain range, with equal points distributed on the south and north slope. In addition, distances between the two adjacent points on the mountain range were relatively equal. Altitudes of the points were extracted from ASTER GDEM V2 with the resolution of 30 m.

Structural equation modeling (SEM) integrates factor analysis and path analysis^25^. It has been widely applied to ecological and environmental studies^19^. The model was constructed by IBM SPSS AMOS 21 software. The model of this study was established by the potential relationships between the five possible variables, i.e., area of Qinghai Lake, temperature, precipitation, evaporation, and snow line. The data of precipitation, evaporation, and temperature were collected from Gangcha meteorological station, which is the nearest meteorological station to Qinghai Lake. The snow line data was collected from Gangshika peak. Then, the stochastic relationship between variables was tested by model reasonableness test.

### Data Availability

The datasets analysed during the current study are available in the China Meteorological Network (http://data.cma.cn/), USGS website (http://landsat.usgs.gov/), and RS Cloud Mart (http://www.rscloudmart.com/en).
